# 2A peptide from ERBV-1 efficiently separates endogenous protein domains in the fission yeast *Schizosaccharomyces pombe*

**DOI:** 10.17912/micropub.biology.000941

**Published:** 2023-09-11

**Authors:** Yuan Ren, Qun Lin, Julien Berro

**Affiliations:** 1 Department of Molecular Biophysics and Biochemistry, Yale University, New Haven, Connecticut, United States; 2 Nanobiology Institute, Yale University, West Haven, Connecticut, United States; 3 Department of Cell Biology, Yale University, New Haven, Connecticut, United States

## Abstract

2A peptides are widely used for polycistronic gene expression from vectors. In contrast, the separation of endogenous genes via 2A peptides has been largely unexplored. We show that in fission yeast *Schizosaccharomyces pombe* , the “cleaving” efficiency of the 2A peptide from ERBV-1 (Equine rhinitis B virus 1) range from ~70% to ~99% for End4 at different insertion sites. Our results suggest a high “cleaving” efficiency as well as considerable variation for using 2A peptide to separate endogenous protein domains in fission yeast. Verification of the “cleaving” efficiency of 2A peptides is advised for its application.

**Figure 1. ERBV-1 2A peptide can be used to separate endogenous protein domains in the fission yeast:**
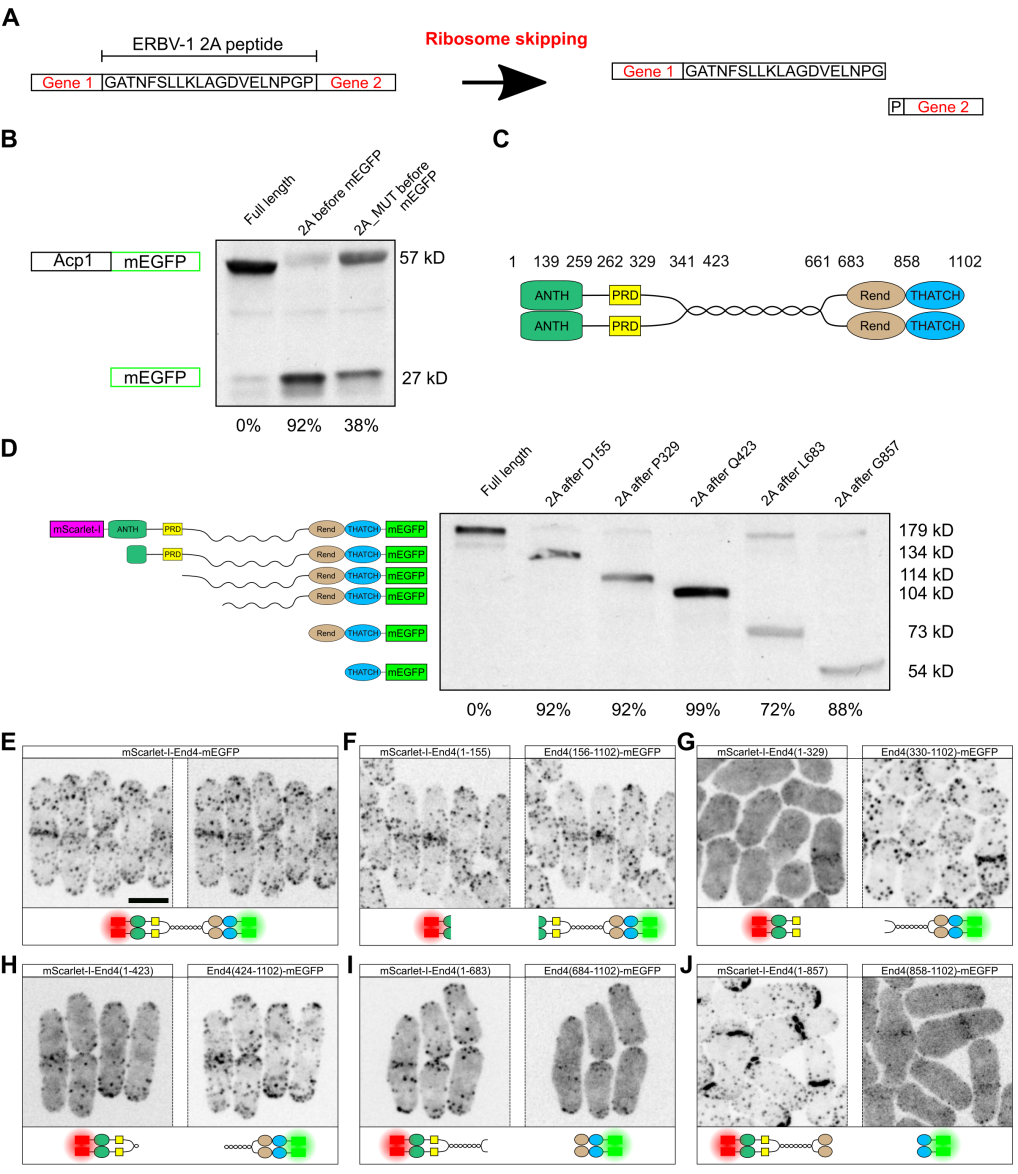
**A.** Schematic for the “cleaving” mediated by 2A peptide. The 2A sequence causes the skipping of a peptide bond by the ribosome between the last two amino acids (glycine and proline), resulting in the expression of two polypeptides from a single mRNA. **B.** Western blot against mEGFP was used to detect the amount of “uncleaved” protein (Acp1-mEGFP, 57 kD) versus the “cleaved” protein (mEGFP, 27 kD). The ratio of “cleaved” protein / (“uncleaved” protein + “cleaved” protein) was used to calculate the “cleaving” efficiency, and was shown below each lane. The last four amino acids of the 2A peptide sequence were mutated into NAGP in the right lane. **C.** Schematic of End4 domain organization. Numbers indicate amino acid sequences. 1-259: ANTH (AP180 N-terminal homology) domain; 262-329: PRD (proline rich domain); 341-423: PolyQ domain; 683-847: Rend (R in End4) domain; 858-1102: THATCH (talin-HIP1/R/Sla2 actin-tethering C-terminal homology) domain. Not drawn to scale. **D.** Western blot against mEGFP was used to detect the amount of “uncleaved” protein (mScarlet-I-end4-mEGFP, 179 kD) versus End4 “cleaved” at different positions. The 2A peptide insertion site was shown at the top of each lane. The “cleaving” efficiency was shown below each lane, calculated as in Fig. 1 **B** . The domain composition of each End4 species (monomer) is shown on the left of the blot, and the expected molecular weight of each fragment is labeled on the right. Results are representative of at least two independent repeats. **E-J** . The cellular localization of wild type End4 ( **E** ) and End4 fragments ( **F-J** ) are shown by imaging through mScarlet-I channel (which labels End4 N-terminus) and mEGFP channel (which labels End4 C-terminus). Fragments of End4 show different localization depending on the insertion site of the 2A peptide. The schematics for End4 fragments are shown below each image. Images are 2D maximum intensity projections of the whole cell. Scale bar in **E** applies to all images: 5 μm.

## Description

2A peptides, also called “2A self-cleaving peptides”, are viral amino acid sequences that mediate the expression of more than one polypeptides from a single mRNA (Ahier & Jarriault, 2014; Lima & Lanza, 2021; Roulston, 2015) . Nascent 2A peptides synthesized from the ribosome are believed to cause ribosome skipping, so that the glycyl-prolyl peptide bond at the end of the 2A peptide is not formed, resulting in two separate polypeptides at 1:1 stoichiometry (Donnelly et al., 2001; Roulston, 2015) ( Figure 1A ). This mechanism circumvents the need for a protease to cleave peptide bonds, and may achieve close to 100% “cleaving” efficiency with only 18-22 amino acids (Kim et al., 2011; Liu et al., 2017; Souza-Moreira et al., 2018) . The term CHYSEL (cis-acting hydrolase element) has been proposed as an alternative name for “self-cleaving” to avoid confusion with protease mediated cleavage (de Felipe, 2004) . Multiple viruses contain 2A peptide sequences in their genomes, and 2A peptides are functional in a wide variety of organisms including those that are not hosts to the viruses, probably due to the conservation of the structure of ribosome (Ahier & Jarriault, 2014; Lima & Lanza, 2021; Tang et al., 2009) . 2A peptides are now routinely used for polycistronic gene expression for cultured cells and in living organisms, gaining popularity over other approaches such as multiple vector transformation, dual promoters or Internal Ribosome Entry Site (IRES). Typically, a 2A peptide, or multiple 2A peptides, are sandwiched by genes on an expression vector to drive the expression of fluorescent tags, protein subunits or other peptide-based functional components (Gao et al., 2018; Lima & Lanza, 2021) . Because 2A peptide offers a “one-shot” solution to remove a peptide bond, we see it as an attractive approach to physically separate the domains of endogenous proteins. This is especially useful when studying the transmission of mechanical forces by protein complexes, where the physical connection between protein domains are indispensable for force transmission (Ren & Berro, 2022) . However, the use of 2A peptides for endogenous proteins at the genomic loci has not been well documented, and their “cleaving” efficiency out of an expression vector is not known.

We first verified the functionality of a 2A peptide sequence before the mEGFP tag. We used the 2A peptide from ERBV-1 because previous analyses in *Saccharomyces cerevisiae* indicates that ERBV-1 2A peptide has the highest “cleaving” efficiency among 22 viral 2A sequences (91% from a plasmid reporter) (Souza-Moreira et al., 2018) . For comparing “cleaving” efficiencies, we used Western blot against mEGFP to visualize different protein fragments. Indeed, insertion of ERBV-1 2A peptide sequence between Acp1 and mEGFP at acp1 gene locus resulted in the “cleaving” of mEGFP with 92% efficiency ( Figure 1B ). Mutation in the ERBV-1 2A peptide sequence, which was predicted to impair ribosome skipping, reduced the “cleaving” efficiency to 38% ( Figure 1B ). These results indicate that 2A peptide could be used to drive polycistronic gene expression from fission yeast genome with a comparable efficiency as in vectors.

We next inserted ERBV-1 2A peptide sequence into end4 locus at five different sites. end4 codes for an endocytic protein, End4, which contains multiple domains and connects the actin cytoskeleton to the plasma membrane during clathrin-mediated endocytosis (CME) (Iwaki et al., 2004; Ren & Berro, 2022; Wesp et al., 1997) . The starting and ending positions of each domain are labeled according to experimental data and bioinformatics ( Figure 1C ) (Abella et al., 2021; Baggett et al., 2003; Garcia-Alai et al., 2018; Gottfried et al., 2010) . By tagging the C-terminus of End4 with mEGFP, and inserting ERBV-1 2A peptide sequence into different positions, the “cleaved” End4 fragment and the “uncleaved” full-length End4-mEGFP can be resolved by blotting against the mEGFP tag ( Figure 1D ). Quantification of the band intensity demonstrate a “cleaving” efficiency ranging from 72% to 99%. End4 fragments within the same cell can be simultaneously visualized by dual-color fluorescence imaging, where the difference in the subcellular localization of End4 fragments confirmed the physical separation of End4 domains ( Figure 1E -J). Collectively, our results indicate that the 2A peptide from ERBV-1 can be used in fission yeast genomic loci to physically disconnect protein domains.

The “cleaving” efficiency from our biochemical assay only compares the ratio of protein fragments that contain the C-terminal mEGFP, and cannot be used to measure the stoichiometry of “cleaved” protein fragments. Our assay was also unable to determine if the apparent variation of “cleaving” efficiency is due to changes at the mRNA level (e.g. mRNA structure) or at the protein level (e.g. protein stability). Compared with using 2A peptide in expression vectors, the insertion of 2A peptide sequence into endogenous genes calls for additional controls because the protein fragments produced by 2A peptide may not fold properly. We do not expect this to be an issue for the genes used in this study, because the insertion sites are either after the entire coding sequence ( Figure 1B ), or guided by structural information (either published or predicted) (Lock et al., 2018) , verified by dual color-imaging ( Figure 1E -J) and consistent with TEVp (Tobacco Etch Virus protease) mediated protein cleavage at the same positions in End4 ( Figure 1D ) (Ren et al., 2021; Ren & Berro, 2022) . For genes with little information or in situations where complete cleaving or 1:1 stoichiometry is critical, it is advised to check the abundance and localization of both fragments flanking the 2A sequence.

## Methods

Yeast strains and media

*S. pombe* strains were made via CRISPR/Cas9 mediated genome editing as shown in (Fernandez and Berro 2016), and all edited gene sequences were confirmed by colony PCR and sequencing. *S. pombe* cells were grown in YE5S (Yeast Extract supplemented with 0.225 g/L of histidine, uracil, adenine, lysine and leucine), at 32 °C on 200rpm shaker.

Electrophoresis and Western blot

Equal number of cells were pelleted and lysed mechanically on BeadBlaster (Millipore, Z742475) in ice-cold lysis buffer (50 mM Tris-HCl, pH=7.4, 150 mM NaCl, 0.5% NP-40, 1 mM DTT with complete protease inhibitor tablet (Millipore, 11697498001)). Samples were boiled in SDS-containing sample buffer before being loaded into an SDS-polyacrylamide gel under reducing conditions. After electrotransfer, the membrane was incubated with an anti-GFP antibody (custom made in Mariappan Lab), followed by a secondary antibody incubation. The protein bands were visualized using chemiluminescence.

Confocal Imaging

Cells were imaged on gelatin pads (25%) on glass slides at room temperature. Slides were mounted on a Nikon TiE inverted microscope (Nikon, Tokyo, Japan) with a CSU-W1 Confocal Scanning Unit (Yokogawa Electric Corporation, Tokyo, Japan) and imaged through a CFI Plan Apo 100X/1.45NA Phase objective (Nikon, Tokyo, Japan). An iXon Ultra888 EMCCD camera (Andor, Belfast, UK) was used for image acquisition. mScarlet-I strains were excited with a 561-nm argon-ion laser and filtered with a single band pass filter (575/25). mEGFP strains were excited with a 488-nm argon-ion laser and filtered with a single band pass filter (510/25). Fluorescence signals were collected by taking 21 consecutive optical sections each with 0.5µm thickness to cover the whole cell, and 2D maximum intensity projected images were created by the Fiji distribution of ImageJ (Schindelin *et al.* 2012).

## Reagents

**Table:** 

Name	Genotype	Used in	Source
SpJB366	acp1 -mEGFP fex1Δ fex2Δ ade6-M216 his3-D1 leu1-32 ura4-D18 h-	1B , left lane	This study.
SpJB552	acp1 -ERBV-1-20AA-mEGFP fex1Δ fex2Δ ade6-M216 his3-D1 leu1-32 ura4-D18 h-	1B , middle lane	This study.
SpJB584	acp1 -ERBV1-20AA_NAGP-mEGFP fex1Δ fex2Δ ade6-M216 his3-D1 leu1-32 ura4-D18 h-	1B , right lane	This study.
SpJB561	mScarlet-I- end4 -mEGFP fex1Δ fex2Δ ade6-M216 his3-D1 leu1-32 ura4-D18 h-	1D , 1st lane; E	(Ren et al., 2021)
SpJB686	mScarlet-I- end4 -D155-ERBV1-20AA-mEGFP fex1Δ fex2Δ ade6-M216 his3-D1 leu1-32 ura4-D18 h-	1D , 2nd lane; F	This study.
SpJB693	mScarlet-I- end4 -P329-ERBV1-20AA-mEGFP fex1Δ fex2Δ ade6-M216 his3-D1 leu1-32 ura4-D18 h-	1D , 3rd lane; G	(Ren et al., 2021)
SpJB796	mScarlet-I- end4 -Q423-ERBV1-20AA-mEGFP fex1Δ fex2Δ ade6-M216 his3-D1 leu1-32 ura4-D18 h-	1D , 4th lane; H	This study.
SpJB872	mScarlet-I- end4 -L683-ERBV1-20AA-mEGFP fex1Δ fex2Δ ade6-M216 his3-D1 leu1-32 ura4-D18 h-	1D , 5th lane; I	This study.
SpJB577	mScarlet-I- end4 -G857-ERBV-1-20AA-mEGFP fex1Δ fex2Δ ade6-M216 his3-D1 leu1-32 ura4-D18 h-	1D , 6th lane; J	(Ren et al., 2021)
